# Overexpression of cry1c* Enhances Resistance against to Soybean Pod Borer (*Leguminivora glycinivorella*) in Soybean

**DOI:** 10.3390/plants13050630

**Published:** 2024-02-25

**Authors:** Qingxi Fang, Yingxue Cao, Thinzar Hla Oo, Chuang Zhang, Mingyu Yang, Yuecheng Tang, Meizi Wang, Wu Zhang, Ling Zhang, Yuhong Zheng, Wenbin Li, Fanli Meng

**Affiliations:** 1 College of Agriculture, Northeast Agricultural University, Harbin 150030, China; qingxifang@126.com (Q.F.); thinzarhlaoo233@gmail.com (T.H.O.); zhang2227508002@163.com (C.Z.); yangmingyu0723@outlook.com (M.Y.); tangyuecheng0116@163.com (Y.T.); wangmeizi0502@163.com (M.W.); 2 Northeast Institute of Geography and Agroecology, Chinese Academy of Sciences, Harbin 150081, China; caoyingxue1993@outlook.com; 3 Heihe Branch of Heilongjiang Academy of Agricultural Sciences, Heihe 164300, China; guoguo_zw@163.com; 4 Jilin Academy of Agricultural Sciences, China Agricultural Science & Technology Northeast Innovation Center, Changchun 130033, China; zly_jaas@126.com

**Keywords:** soybean, transgenic soybean, soybean pod borer, cry1c*

## Abstract

Soybean [*Glycine max* (L.) Merr.], an essential staple food and oil crop worldwide, boasts abundant vegetable proteins and fats beneficial for both human and animal consumption. However, the soybean pod borer (*Leguminivora glycinivorella*) (SPB) stands as the most destructive soybean insect pest in northeast China and other northeastern Asian regions, leading to significant annual losses in soybean yield and economic burden. Therefore, this study aims to investigate the introduction of a previously tested codon-optimized *cry1c* gene, cry1c*, into the soybean genome and assess its effect on the SPB infestation by generating and characterizing stable transgenic soybeans overexpressing cry1c*. The transgenic soybean lines that constitutively overexpressed cry1c* exhibited a significant reduction in the percentage of damaged seeds, reaching as low as 5% in plants under field conditions. Additionally, feeding transgenic leaves to the larvae of *S. exigua*, *S. litura*, and *M. separta* resulted in inhibited larval growth, decreased larval body weight, and lower survival rates compared to larvae fed on wild-type leaves. These findings showed that the transgenic lines maintained their resistance to SPB and other lepidopteran pests, especially the transgenic line KC1. Southern blotting and genome-wide resequencing analysis revealed that T-DNA integration occurred as a single copy between loci 50,868,122 and 50,868,123 of chromosome 10 in the transgenic line KC1. Therefore, the transgenic line KC1, overexpressing high levels of cry1c* in leaves and seeds, holds strong potential for commercial use in the integrated management of SPB and other lepidopteran pests.

## 1. Introduction

Soybean [*Glycine max* (L.) Merr.], an important crop globally, provides high-quality vegetable proteins and oils for human and animal consumption. It currently contributes approximately 3.3% to the global human calorie intake, ranking first among the four major crops worldwide, including wheat, rice, maize, and potato [1]. The growing population and rising consumption patterns drive an ever-growing demand for soybeans [2]. However, soybean yield faces significant constraints owing to the diversity of biotic stressors such as bacteria, fungi, insects, and other organisms. Of these stressors, insect pests pose particularly challenging biotic stress, presenting challenges in prevention while demanding huge inputs of human resources, material, and financial resources. Pest damage not only decreases the market quality of soybeans but also significantly lowers the yield [3]. Among the diverse pests, damage caused by lepidopterans stands out as particularly severe [4,5,6,7,8]. It has been reported that approximately 44% of soybean yield reduction in the southeast of the United States is attributed to lepidopteran pests [9,10]. Similar instances of such pest damage have also been documented in China. Previous studies have identified *Leguminivora glycinivorella Matsumura*, *Spodoptera litura*, *Spodoptera exigua*, and *Mythimna separata* [11] as the primary lepidopteran pests responsible for reducing soybean yields by 15%. Approximately half of the plants will be affected, resulting in substantial economic losses annually.

SPB (*Leguminivora glycinivorella*) constitutes a significant threat to soybean crops in China and other northeastern Asian countries [12,13]. During the summer months, female SPBs oviposit on soybean plants, with the resulting larvae burrowing into soybean pods, which provide an optimal environment for their development. Consequently, pods damaged by SPB larvae exhibit decreased seed quality and yield [14,15]. Several strategies have been employed in field management processes to mitigate SPB damage. Presently, pesticide-based control strategies remain the predominant methods for SPB control. However, unlike other pests, SPBs have a longer life cycle and the potential for recurrent outbreaks [16], rendering control efforts challenging. Moreover, excessive pesticide application places significant strain on the environment, contradicting principles of sustainable development. Furthermore, widespread and repetitive pesticide use accelerates the development of pesticide resistance [17]. Consequently, other control strategies, such as biological control [18] and pheromone-based methods [19], have gained traction in recent decades. However, these control methods face limitations comparable to chemical control. Developing transgenic soybeans with SPB resistance is the most efficient, economical, and environmentally friendly way to overcome the constraints associated with the strategies mentioned above and manage this type of insect pest.

*Bacillus thuringiensis* (*Bt*), discovered in the soil, is a Gram-positive spore-forming bacterium. *Bt* encompasses a diverse family of subspecies found in various habitats [20], with 72 antigenic groups identified [21,22]. More than 900 toxin genes encoding diverse entomopathogenic proteinaceous toxins have been identified and characterized in *Bt* strains globally. Most of these toxins are produced as parasporal inclusions during sporulation. With its entomopathogenic properties and toxin genes, *Bt* has been widely used to enhance pest resistance in crops. Since 1996, approximately 200 genetically modified (GM) varieties and lines of *Bt* crops from eight plant species (cotton, corn, eggplant, potato, poplar, rice, soybean, and tomato) have been developed and approved for commercial release [20]. Soybean, as an essential legume, has been the subject of research on pest-resistant varieties for several decades. For example, overexpressing the *Cry8*-like gene in the soybean cultivar Jinong28 enhances the resistance of transgenic soybeans against *Holotrichia parallela* [23]. Another study found that transgenic soybeans expressing double-stranded RNA of SpbP0 exhibit greater resistance to SPB than wild type (WT) plants [14]. In 2015, approximately 75 million ha of *Bt* transgenic crops were planted and harvested, leading to a reduction in pesticide usage in agricultural fields. According to reported data, a reduction in pesticide utilization of more than 583 million kg was achieved between 1996 and 2014 [24].

*Cry* and *Cyt* are two different types of insecticidal crystal proteins (ICPs) derived from *Bt*. They are classified based on their amino acid homology. *Cry* is a toxin gene isolated from *Bt* and its corresponding proteins demonstrate toxic effects on target organisms under verifiable experimental conditions. The primary mechanisms of action of Cry toxins involve the lysis of midgut epithelial cells through insertion into the target membrane and pore formation [25]. Furthermore, these toxins display high specificity for their target insects and pose no threat to humans, plants, and vertebrates [26]. Therefore, Cry toxins offer a promising means of controlling insect pests in agricultural settings. For instance, approximately 60 GM crops utilize the *cry3A*, while 34 GM crops utilize the *cry34Ab1-cry35Ab1* genes to develop pest-resistant varieties against coleopteran pests [20,27]. Furthermore, the successful expression of *Cry10Aa* in cotton flower bud tissue demonstrates strong resistance against the cotton boll weevil (CBW), suggesting potential commercial utility in future integrated CBW management [28].

Moreover, these proteins are biologically inactive in their native forms. Upon ingestion, they undergo solubilization and enzymatic processing in the insect guts, leading to the formation of an increasingly active soluble toxin capable of binding with midgut epithelial receptors [29]. Previous studies have reported that CryI toxins are effective against lepidopteran pests, leading to their widespread utilization in pest control across several countries, including China, America, Brazil, Argentina, and Japan. 

Therefore, this study aims to investigate the development and evaluation of cry1c* transgenic soybean lines for resistance against SPB and other lepidopteran pests. This involves introducing the cry1c* gene into the genome of the soybean variety Kennong18 (KN18) and assessing the efficacy of target and marker genes in the transgenic lines in controlling pest populations through three consecutive generations (T1 to T3), quantitative real-time polymerase chain reaction (qRT-PCR), in-planta feeding bioassays, genetic stability testing, and protein analysis. The results of in-planta feeding bioassays clearly demonstrated that feeding on cry1c* transgenic soybean leaves leads to severe mortality in *S. exigua*, *S. litura*, and *M. separta*. Importantly, the overexpression of cry1c* does not alter the phenotype or agronomic traits compared to KN18 under field conditions. Using SDS-PAGE and enzyme-linked immunosorbent assay (ELISA), approximately 70 kDa protein representing cry1c* is obtained and quantitatively analyzed. The results indicate higher levels of cry1c* enrichment in leaves, pods, and seeds at the V2, R3, R6, and R8 developmental stages. Overall, the transgenic lines overexpressing cry1c* developed in this study enable resistance against SPB and hold strong potential as valuable germplasm for commercial use in integrated SPB and other lepidopteran pest management. 

## 2. Results

### 2.1. Development of cry1C* Transformed Soybean

**Figure 1:**
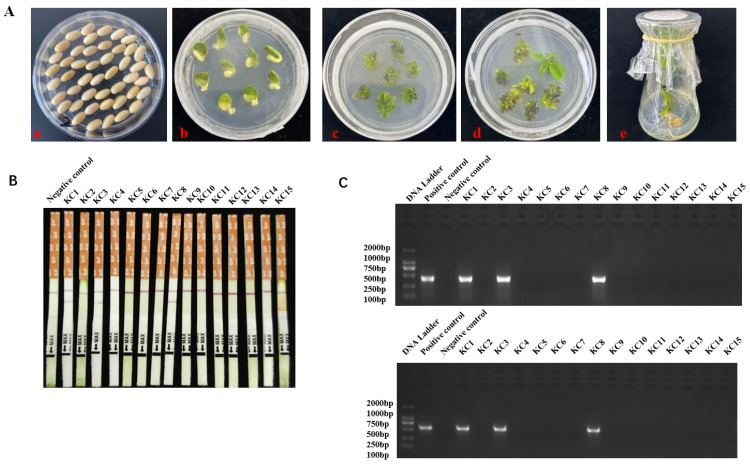
Developmental stages of cry1c* transformed soybean. (**A**) Overview of soybean transformation processes.(**a**) Germinated seeds; (**b**) Explants after 3 days of co-culture; (**c**) Shoot induction; (**d**) Shoot elongation; (**e**) Explants growing in medium. (**B**) Detection of phosphinthricin acetyltransferase (PAT) protein using LibertyLink test strips. (**C**) PCR test electrophoresis gel featuring *Bar* and cry1c* tests. The upper panel displays the *Bar* test, while the lower panel shows the cry1c* test.

To develop transgenic soybeans, the construct pCAMBIA1300-cry1C* was created and introduced into the KN18 genome using the cotyledonary node transformation strategy, which Agrobacterium mediated. Overall, approximately 16,000 soybean cotyledonary nodes were transformed. After a series of processes, including germination, co-culture, shoot induction, shoot elongation, and rooting (Figure 1A), 15 Bar-resistant soybean plantlets were obtained. LibertyLink test strips revealed the presence of bands related to phosphinthricin acetyltransferase (PAT) in transgenic soybean plants. While the control band was observed in both transgenic and non-transgenic plants, the PAT protein bands were exclusively present in transgenic soybean plants and absent in non-transgenic ones. These findings indicate the successful expression of the PAT protein in the three resistant soybean plantlets, KC1, KC3, and KC-8 (Figure 1B). Transgene insertion was confirmed through conventional polymerase chain reaction (PCR) using specific primers (Table S1) for the *Bar* selectable marker and cry1c* genes, respectively (Figure 1C). The expected 403 bp and 603 bp *Bar* and cry1c* fragments were amplified only from genomic DNA isolated from the KC1, KC3, and KC8 plantlets (Figure 1C). Subsequently, the three transgenic lines were transplanted into plastic pots containing approximately 25 kg of soil and maintained under greenhouse conditions until seed collection.

### 2.2. Confirmation of Genetic Stability in Transgenic Soybean Lines

**Figure 2:**
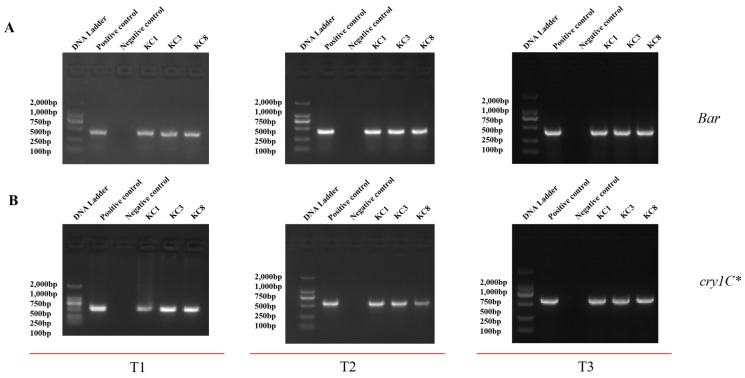
Genetic stability analysis across consecutive T1 to T3 generations. (**A**) PCR test electrophoresis gel for *Bar* across generations T1 to T3. (**B**) PCR test electrophoresis gel for cry1c* across generations T1 to T3.

Ensuring the stable insertion of target and marker genes into the genome is important for seeking commercial release. To assess the genetic stability of these genes in transgenic lines KC1, KC3, and KC8, PCR reactions were conducted. These reactions amplified 403 bp and 603 bp fragments corresponding to the *Bar* and cry1c* genes, respectively, using specifically designed primers. The PCR analysis revealed the presence of both cry1c* and *Bar* genes in all transgenic plants. Furthermore, it demonstrated that these genes were stably inserted into the genomes of all three soybean lines. The soybean events exhibited homozygosity for the inserted cry1c* and *Bar* genes (Figure 2A,B).

### 2.3. Analysis of cry1C* mRNA Expression Levels

**Figure 3:**
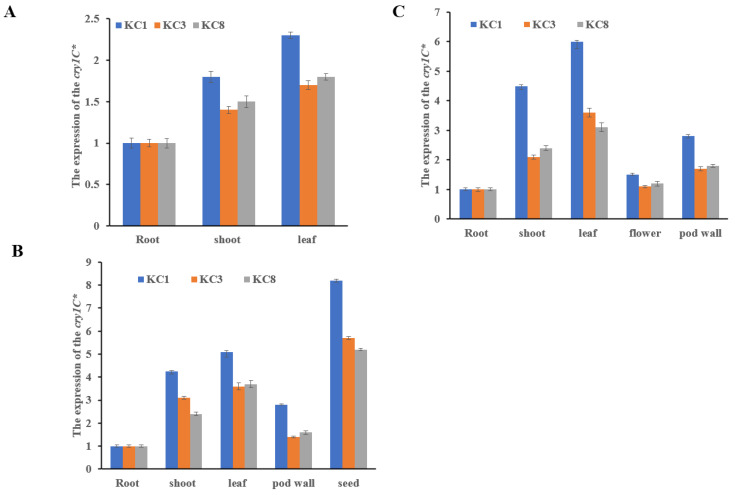
Expression level of cry1c* at different developmental stages. The expression level of cry1c* was quantified using real-time qPCR. (**A**) V2 developmental stage; (**B**) R3 developmental stage; (**C**) R6 developmental stage. The numbers on the Y-axis represent the fold change in relative expression level analyzed using the 2^-ΔΔCt method. Error bars indicate SD.

The cry1c* gene expression levels were analyzed at different developmental stages (V2, R3, and R6) and in different tissues (root, shoot, leaf, flower, pod wall, and seed) across three independent transgenic lines (KC1, KC3, and KC8) using qRT-PCR. The analysis revealed a higher relative expression of cry1c* mRNA in the shoots and leaves at the V2 stage (Figure 3A). Similarly, a significantly high expression level of cry1c* mRNA was observed in the shoots and leaves at the R3 stage (Figure 3B). At the R6 stage, the expression levels of cry1c* mRNA were higher in the seeds than in the shoots and leaves (Figure 3C). The cry1c* expression levels in the shoots and leaves were higher than that in the pod wall on average, while the expression in the roots was the lowest. These findings indicate that cry1c* is highly expressed in shoots, leaves, and seeds across transgenic lines. However, the expression levels of cry1c* vary significantly across transgenic lines, with the KC1 line showing the highest expression level compared to other lines.

### 2.4. Field Simulation for Evaluating Resistance to SPB

**Figure 4:**
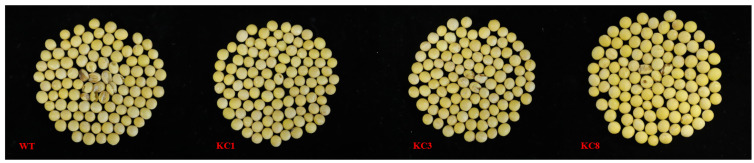
Analysis of spotted pod borer (SPB) feeding damage on seeds in transgenic lines and control. One hundred seeds from each transgenic line and WT were collected, and the ratio of SPB feeding damage was calculated. The analysis was conducted in three replicates.

In this study, the T1, T2, and T3 generations of transgenic soybean plants (specifically lines KC1, KC3, and KC8), along with KN18 plants, all covered by a plastic mesh cage, were evaluated for SPB feeding damage. The plants, at their R5 developmental stage, were exposed to extreme biotic stress by 30 adult SPB m^−2^ infection. This setup aimed to replicate heavy field infection conditions and to give a real challenge of SPB infestation to the soybean plants. The field assay results from T1 to T3 revealed that the transgenic lines were resistant to SPB and had fewer damaged seeds than KN18 plants (Figure 4). Moreover, variations in resistance were observed among the transgenic lines. The transgenic line KC1 demonstrated high resistance to SPB (Table 1), with seed samples from T1 to T3 containing only 0.31%, 1.72%, and 0.54% of damaged seeds, respectively.

### 2.5. In-Planta Feeding Bioassays against Lepidopteran Larvae

**Figure 5:**
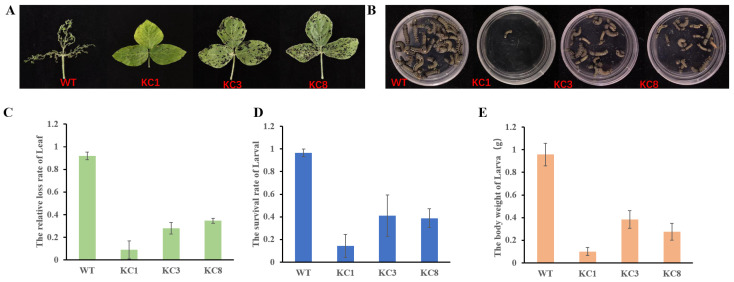
In-planta feeding bioassays of *S. exigua*. (**A**) Phenotypic depiction of feeding situation by *S. exigua* larvae; (**B**) survival of *S. exigua larvae* after 15 days of feeding; (**C**) relative loss rate of leaves consumed by *S. exigua* larvae; (**D**) survival rate of *S. exigua* larvae; and (**E**) the body weight of *S. exigua* larvae.

Bioassays were conducted to assess the resistance of transgenic lines overexpressing cry1c* against lepidopteran larvae such as *Spodoptera exigua (Hübner)*, *Spodoptera litura (Fabricius)*, and *Mythimna separta (Walker)*, which feed on soybean leaves. The first-instar larvae were exposed to cry1c* transgenic soybean leaves at the R3 stage to observe the rate of leaf loss, larval development, and survival. After 3 days of larval feeding, significant differences in leaf loss rate were observed. *Spodoptera exigua* consumed the entire leaf of WT, resulting in a relative loss rate of 91.85%. Conversely, larvae feeding on transgenic lines only created shallow holes on the leaves, with relative loss rates ranging from 0.08% to 24.65% (Figure 5A,C). Furthermore, the survival rate of larvae feeding on transgenic leaves was significantly lower, especially in KC1, where <20% survival rate was observed after 15 days of feeding, compared to 75.67% in the WT. The larvae that fed on transgenic soybean leaves remained small and stunted, whereas those that fed on WT leaves developed completely (Figure 5B,D). The larvae that fed on WT leaves were significantly heavier than those that fed on transgenic soybean leaves (Figure 5E). Similar results were observed in the feeding bioassays of *S. litura* and *M. separta* (Figures S1 and S2). These findings clearly demonstrate that feeding on cry1c* transgenic soybean leaves led to the mortality of *S. exigua*, *S. litura*, and *M. separta*.

### 2.6. Effect of cry1C* Overexpression on Soybean Yield and Seed Quality

To assess any potential effect on the phenotype of cry1c* transgenic soybeans, all transgenic lines and WT plants were planted in the field. The results showed no significant differences in agronomic traits, including protein and oil contents, between the transgenic lines and the WT (Table 2). This suggests that the transgene had no discernible effect on the soybean phenotype or the tested agronomic traits.

### 2.7. Determination of Transgene Copy Number and Insertion Sites

**Figure 6:**
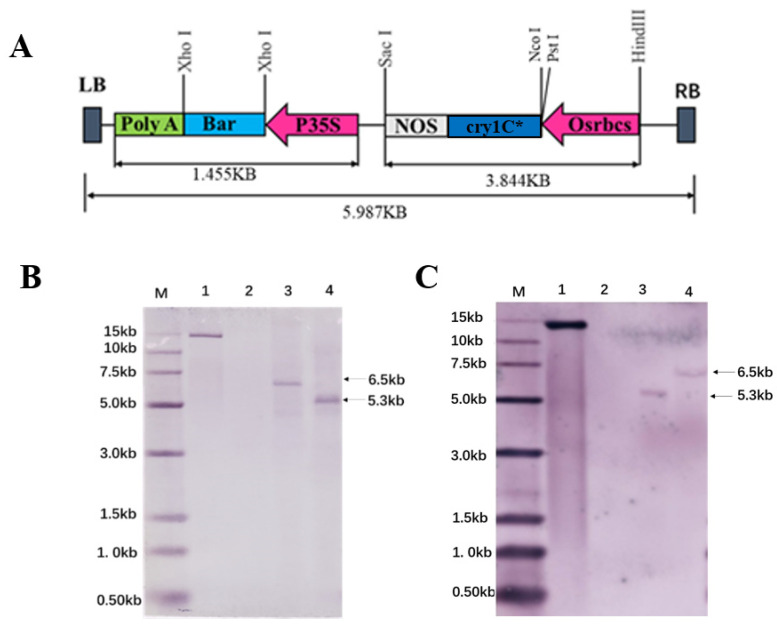
Screening copy number of transgenic line KC1. (**A**) Plasmid map indicating the locations of *Bar* and cry1c*; (**B**) Southern blot for Bar copy number in KC1 (M, DNA Ladder; lanes 1, 2, 3, and 4 loaded with positive plasmid, negative control, isolated DNA digested with NcoI, and isolated DNA digested with PstI, respectively); (**C**) Southern blot for cry1c* copy number in KC1 (M, DNA Ladder; lanes 1, 2, 3, and 4 loaded with positive plasmid, negative control, isolated DNA digested with PstI, and isolated DNA digested with NcoI, respectively).

**Figure 7:**
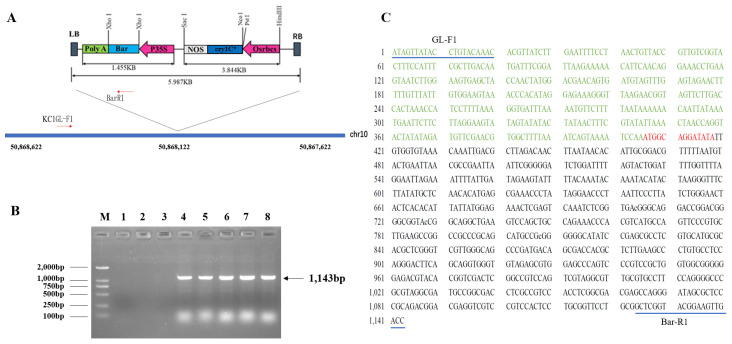
Transgene insertion sites in the transgenic line KC1. (**A**) Genome location of transgene insertion in the transgenic line; (**B**) electrophoresis gel depicting PCR test with specific primers. M, DNA Ladder; 1, 2, 3, 4, 5, 6, 7, and 8 are negative control, distilled water control, plasmid sample, and five T3 individual plants of KC1, respectively; (**C**) confirmation of endogenous genes completeness. The soybean genomic sequence is highlighted in green; unknown insertion sequence is labeled in red; the left flanking region sequence of T-DNA is highlighted in black.

According to findings from the field and in planta efficacy studies, the transgenic soybean line KC-1 showed high resistance to SPB, *S. exigua*, *S. litura*, and *M. separta*. Further screening was conducted in the T3 generation using Southern blot analysis. The genome of the transgenic soybean line KC-1 was digested with NcoI and PstI enzymes, revealing that the cry1c* and *Bar* genes were located in the same region (Figure 6A). With the cry1c* and *Bar* gene hybridization probe, a stable signal indicating the integration of the plasmid segment containing the cry1c* and *Bar* gene into the transformed plant genome presented as 5.3 and 6.5 Kb bands, respectively. This suggests the creation of a single-copy transgene (Figure 6B,C). The plasmid pCAMBIA1300-cry1c* showed a band size of 12.0 Kb, whereas DNA isolated from non-transformed plants did not yield any hybridization signal. The transgene insertion sites in the transgenic soybean line KC-1 were successfully identified using genome-wide resequencing technology. These insertion sites were mapped to 50,868,122 loci on chromosome 10. Specific primers were designed according to the *Bar* gene sequence and genomic sequence adjacent to the insertion site (Figure 7A). The expected 1143 bp fragment was amplified exclusively from genomic DNA isolated from KC1 plants. The PCR results showed the integration of T-DNA as a single copy into the loci of chromosome 10, ranging from 50,868,122 to −50,868,123 (Figure 7B), without causing the knockout or knockdown of endogenous genes (Figure 7C). However, an unknown 13 bp insertion was observed between the 50868123 loci and the T-DNA sequence.

### 2.8. Successful Expression of cry1C* Protein in Transgenic Soybeans 

SDS-PAGE analysis revealed approximately 70 kDa protein, indicating cry1c* presence in various tissues, including the root, shoot, leaf, flower, and pod tissues, during the R3 developmental stage of the transgenic line KC1. This finding was further confirmed via Western blotting (Figure S3). To assess relative protein levels across different developmental stages (V2, R3, and R6) and tissues, an enzyme-linked immunosorbent assay (ELISA) was employed to quantify cry1c* protein in the transgenic line KC1. The protein content analysis revealed significant variations in different tissues and developmental stages of the transgenic soybean line KC1. At the V2 stage, the highest protein content of 4.81 ± 0.43 µg g^−1^ fresh weight was observed in the leaves, whereas the lowest protein content of 0.26 ± 0.15 µg g^−1^ was found in the roots. At the R3 stage, the pod exhibited the highest protein content (18.4 ± 0. 81 µg g^−1^). Subsequently, at the R6 and R8 stages, the seeds displayed the highest cry1c* protein content, with values of 14.74 ± 0.67 µg g^−1^ and 12.43 ± 0.66 µg g^−1^, respectively (Table 3).

## 3. Discussion

Overall, three transgenic lines, KC1, KC3, and KC8, were developed, each overexpressing the toxin gene cry1c*, demonstrating high resistance against lepidopteran pests. The Cry toxin is considered as a parasporal inclusion protein derived from *Bt* that exhibits toxic effects [25]. As an important pore-forming toxin (PFT), Cry undergoes secretion as a water-soluble protein and it is subsequently inserted into the host membrane after conformational alterations. When ingested by pests, the Cry toxin undergoes solubilization in their midgut. Subsequently, it is activated by midgut proteases. This activated form binds to specific receptors on the cell membrane of pests, resulting in cellular disruption and death. Based on this mechanism, various transgenic soybean varieties expressing *the Bt* gene have been developed, resulting in increased resistance to critical pests [30,31]. Pest-resistant transgenic breeding began relatively early compared to other breeding traits, yielding several varieties. For example, the overexpression of *CryIAc* derived from Jack-Bt increased its resistance to four different types of lepidopteran pests [5], making *CryIAc* a widely applied *Bt* gene in pest-resistant soybean breeding efforts [7,32].

The introduction of the modified cry1c* in rice significantly increases resistance against the *Chilo suppressalis walker* and *Tryporyza incertulas walker*. In this study, the modified cry1c* gene was integrated into the genome of KN18, a variety known for its high oil content in Heilongjiang Province, China. The analysis of the resulting transgenic line KC1 revealed the highest expression of cry1c* protein in immature seeds, while the lowest expression was observed in the husks of pods. This high expression level correlates with high resistance to SPB larvae that feed on transgenic immature seeds. The cry1c* protein content in leaves was relatively high, suggesting resistance to lepidopteran pests such as *S. exigua* and *M. separate*. Compared to other tested tissues, it was observed that the cry1c* expression was at its lowest, both transcriptionally and translationally, in the roots. This indicates that the significant resistance of the transgenic lines against SPB and other tested lepidopterans is primarily ensured by the leaf- and seed-specific high expression of cry1c*. These pests undergo almost the entire life cycle from vegetative to reproductive growth. These findings suggest that the expression of codon-optimized cry1c* in soybeans enhances the resistance of transgenic plants against lepidopterans. Regarding lepidopteran resistance, cry1c* and CryIAc display similarly strong abilities. However, the recognition sites of the two proteins in pests are entirely different [33], suggesting no risk of cross-pest resistance between the development of transgenic soybean expressing cry1c* and the commercial transgenic soybean variety expressing the *CryIAc* gene.

Trade-offs between different agronomic traits pose significant constraints on crop breeding. These constraints include the dilution effect on plant nutrition and grain quality, crop yield, immunity, and negative correlations among yield traits. Achieving a balance between these complex trade-offs is important for enhancing crop genetic improvement [34]. KN18, a cultivated variety in Heilongjiang Province, contains high oil content, which ranks among the most critical quality traits. In this study, KN18 served as the transgenic receptor, and three transgenic lines were developed, showing enhanced SPB resistance. Upon analyzing both oil and protein content, no significant difference in these traits was observed between KN18 and the transgenic lines. This suggests that overexpressing cry1c* did not affect the main quality of the transgenic line. These transgenic lines not only demonstrated SPB resistance but also maintained the original high oil content, balancing the two crucial characteristics. Consequently, they offer valuable germplasm for advancing breeding practices. 

Since its discovery, *Bt* has been widely used and plays a crucial role in agricultural production. This trend of increased *Bt* utilization is expected to persist in the future. Despite numerous studies investigating *Bt* structure, toxin genes, and associated features in the past few decades, a big knowledge gap still persists concerning the detailed understanding of the physiological and biochemical pathways alongside the mode of action of *Bt.* This knowledge deficit results from the limited information available in functional genomics, metabolomics, and proteomics [20]. With the advancement of next-generation sequencing (NGS) technology, complex studies and analyses can be conducted more seamlessly, enabling efficient *Bt* exploration.

Pesticide control is a prevalent strategy in modern agriculture aimed at managing pests and protecting food production to meet global food demand. Datasets report that approximately 2 million tons of pesticides are annually applied to fields globally to ensure crop yield and quality. However, as of 2020, global pesticide usage has escalated to 3.5 million tons [35]. The World Health Organization (WHO) reports a rapid increase in pesticide usage in southeast Asian countries. Pesticide importation has increased by 61%, 55%, and 10% in Cambodia, Laos, and Vietnam, respectively [36]. The overuse of various pesticides in agricultural practices contributes to air, water, soil, and overall ecosystem contamination [37], which may have serious hazardous effects on living beings. According to a global database on pesticide applications, approximately 64% of global agricultural land is at risk of pesticide pollution resulting from one or more active ingredients released by pesticides [37]. 

The application of pesticides in agriculture is projected to increase in the future due to population growth and rising food demand [38]. To mitigate environmental pollution caused by pesticides, biotechnology offers an efficient strategy for developing pest-resistant crops. This approach reduces pesticide usage and its associated economic costs. In this study, the transgenic lines KC1, KC3, and KC8, which display high resistance against lepidopteran pests, were developed. These pests are among the most severe in China and northeastern Asian countries, making these transgenic lines valuable for potentially reducing pesticide usage in agricultural fields. Specifically, the transgenic line KC1 stands out as a potential germplasm for breeding pest-resistant breeding soybeans. Incorporating this type of transgenic line into agricultural practices presents a viable approach to promoting sustainable agricultural development [39]. 

## 4. Materials and Methods

### 4.1. Generation of cry1c*-Overexpressing Transgenic Plants

The modified pCAMBIA1300 vector, containing *pBar* as a plant selection marker, served as the backbone for creating transgenic soybeans. The exogenous gene cry1c* gene and the green tissue-specific synthetic promoter Posrbcs were provided by Professor Lin Yongjun of Huazhong Agricultural University [40]. Both the backbone vector and cry1c* gene underwent double digestion at 37 °C for 2 h using restriction enzymes KpnI (Thermo Fisher Scientific, Waltham, MA, USA, Cat# FD0524) and BamHI (Thermo Fisher Scientific, Waltham, MA, USA, Cat# FD0054) before ligating. Subsequently, the cry1c* gene was ligated into the linearized pCAMBIA1300 vector using a T4 DNA ligase kit at 22 °C for 2 h and 16 °C for maintenance (Thermo Fisher Scientific, Waltham, MA, USA, Cat# 15224041). The ligation product was then transformed into commercial competent cells DH5a (Thermo Scientific, Waltham, MA, USA, Cat# EC0112), and the plates were cultured overnight at 37 °C. After Sanger sequencing, the verified pCAMBIA1300-CryIC* plasmid was isolated from the sequenced bacteria using the FastPure Plasmid Mini Kit (Vazyme, Nanjing, China, Cat# DC201-01), following the instructions of the manufacturer. Subsequently, it was transfected into Agrobacterium tumefaciens strain EHA105 using the electroporation method with an electroporator (Bio Rad, Hercules, CA, USA, 1652100). Following that, the soybean variety KN18 underwent transformation using the cotyledonary node-based method with Agrobacterium strain EHA105. Transgenic plants were then selected based on their tolerance using phosphinothricin (PPT; Sigma, St. Louis, MO, USA, 45520). Rooted PPT-resistant plantlets were subsequently transplanted into soil and grown under greenhouse conditions until maturation. 

### 4.2. Molecular Screening of Transgenic Soybean Lines 

Total DNA from the rooted PPT-resistant plantlets was isolated using a widely used DNA extraction kit (Kangwei Century Biotechnology Co., Ltd., Beijing, China). The integration of transgenes into the soybean genome was confirmed through conventional PCR using specific primers for the *bar* selectable marker and cry1c* gene (Supplemental Table S1). The detailed PCR program consisted of the following steps: initial denaturation at 98 °C for 5 min; denaturation at 98 °C for 10 s; annealing at 58.8 °C for 15 s; extension at 68 °C for 1 min; final extension at 68 °C for 5 min; and maintenance at 12 °C. PCR products were analyzed using agarose gel electrophoresis on approximately 1.8% agarose gel. Additionally, soybean leaves that overexpressed the cry1c* transgene and carried the *bar* selectable marker gene were screened using LibertyLink test strips (ENVIROLOGIX INC, Portland, ME, USA). The T1-T3 generations of the transgenic soybean lines underwent PCR analysis to confirm the presence of cry1c* and *bar* in the transgenic offspring.

### 4.3. Expression Analysis of cry1c* mRNA across Developmental Stages and Tissues

Total RNA was isolated from various tissues at different developmental stages (i.e., V2, R3, and R6) of the transgenic lines using an Invitrogen Plant RNA Purification Reagent kit (Invitrogen, Carlsbad, CA, USA), following the instructions of the manufacturer. Subsequently, the expression of cry1c* mRNA was quantified with quantitative real-time PCR (qRT-PCR). The qRT-PCR analysis was conducted with an Invitrogen One-Step RT-PCR kit (Invitrogen, Cat# 12594025) and an iCycler iQ5 Real-Time PCR Detection System (Bio-Rad, Hercules, CA, USA). *GmActin* (NM 001289231) served as a positive internal control. Each sample comprised three biological replicates with three plants per sample. In this study, the expression of cry1c* in the roots was selected as the calibration sample.

### 4.4. Field Simulation for Evaluating Transgenic Plant Resistance to SPB 

The T1, T2, and T3 generations of three independent transgenic lines (KC1, KC3, and KC8) and the receptor control KN18 plants underwent insect bioassays under field conditions. Field evaluations were conducted in experimental plots at the Northeast Agricultural University. A randomized block design was implemented, with 35 rows in each plot, each row measuring 1 m in length and 60 cm in width, with 7 cm of plant spacing and 17 seeds sown in each row. The seedlings were subjected to the bar strip assay once they grew three compound leaves, and the test result was considered negative if the seedlings could be directly pulled out. Before treatment, a net shed covered the plants, and in early August, transgenic and receptor control plants with developing pods were infested with 30 adult SPB m^−2^, allowing them to freely choose plants to lay eggs, bore pods, and feed on soybean grains. The percentage of damaged seeds (i.e., the number of seeds damaged by SPB divided by the total number of seeds) was calculated post harvest.

The agronomic and seed quality traits of T3 transgenic soybean plants and KN18 plants were collectively evaluated. The seed crude protein concentration (mg kg^−1^) and seed oil concentration (mg kg^−1^) of each line were analyzed using a Grain Analyzer Infratec TM 1241 (FOSS, Hillerød, Denmark). Data were collected from ten seed samples (minimum of 60 g), and the averages were calculated. 

### 4.5. In Planta Feeding Bioassay

T3 seeds of the transgenic lines KC1, KC3, and KC8, as well as those from the WT KN18, were planted in a greenhouse. *S. litura*, *S. exigua*, and *M. separate* larvae were obtained from the company Henan Jiyuan Baiyun Industry Co., Ltd. (Jiyuan, China). To assess the effects of transgenic plants on these pests, 30 first-instar larvae were reared for 15 days on soybean leaves collected from transgenic or WT plants at the R3 stage. The leaf loss rate was evaluated 3 days after the inoculation of first-instar larvae. The body weights and survival rates of *S. litura*, *S. exigua*, and *M. separate* larvae were determined 15 days after feeding on the leaves. 

### 4.6. Copy Number and Insertion Site Estimation of the Transgenic Line KC1

Southern blot analysis was conducted to confirm transgene integration into the soybean genome and to estimate the transgene copy number of the transgenic line KC-1. cry1c* and *Bar* genes were amplified using a PCR-based method with specific primers (Table S1). Genomic DNA was isolated from KC-1 plants using the high-salt CTAB DNA isolation method. Approximately 30 µg of genomic DNA was overnight digested with PstI (Thermo Scientific, Cat# FD0615) or NcoI (Thermo Scientific, Cat# FD0574) restriction enzymes for further analysis. cry1c* and Bar probes were labeled using the DIG High Prime DNA Labeling Kit (Roche Cat. No. 11585614910), according to the instructions of the manufacturer. Hybridization was performed at 42 °C. Washing and visualization were performed according to the manufacturer’s instructions.

Genomic DNA was extracted from mixed samples, including ten T3 plants of the transgenic line KC-1. Whole-genome sequencing was performed by Biomarker Technologies Co., Ltd. (Beijing, China). Using bwa software (version 0.61-r104), the genome sequencing data were compared to LB/RB sequences of pCAMBIA1300-Cry1C*. Sequencing reads located at LB/RB sides were extracted and mapped to the Willams82 reference genome using BLAST (BLAST version 2.8.1+) to obtain the insertion sites.

### 4.7. Assessment of cry1c* Expression Using Western Blot

At the R3 stage, fresh roots, shoots, leaves, flowers, and pods were harvested from the transgenic line KC1 plants and the recipient plant KN18. They were immediately preserved in liquid nitrogen. The ground tissue powder was collected in centrifuge tubes. Each tube received 200 μL of protein extraction solution, which was thoroughly mixed, vortexed for 1 min, and then subjected to an ice bath for 10 min. After this, centrifugation was performed at 4 °C, at 13,000 rpm for 15 min. Subsequently, 80 μL of the supernatant was aspirated into a 1.5 mL centrifuge tube and stored at −20 °C for short-term preservation. The SDS-PAGE gel was divided into two sections: the upper section, containing a 4% concentration polymerized gel, and the lower section, consisting of a 12% concentration separation gel. It was ensured that the electrophoresis solution covered the thin plate between the gel plates and that the solution level outside the glass plates was higher than that of the platinum wires. Samples were added sequentially to the gel wells, and electrophoresis commenced once all samples had been dispensed. After electrophoresis, the proteins were transferred to a polyvinylidene fluoride (PVDF) transfer membrane (Thermo Scientific, Cat# 88518). The transferred PVDF membrane was then placed in a 5% skim milk (BD, Cat# 232100) solution, sealed, and incubated overnight at 20 °C with gentle shaking. Subsequently, the membranes were incubated with antibodies. The primary antibody used was specific to cry1c*. The secondary antibody labeled with HRP was then applied. Finally, the band was detected using a Tanon 5200 Multi-imaging system.

### 4.8. Assessment of cry1c* Expression Using ELISA

Samples were collected at different growth stages, including the seedling (V2), flowering, pod-setting (R2), pod-filling (R6), and maturity stages (R8) of soybean plants from both the KN18 and transgenic strain KC1 generations. Root, leaf, stem, flower, and seed tissues were selected, rapidly frozen in liquid nitrogen, and stored for further analysis. The samples were finely ground into a powder, and 0.05 g of each sample was added to 500 µL of sample extraction solution. After shaking and mixing for 5 min, the mixture underwent centrifugation at 4000 rpm for 3 min, and 100 µL of the supernatant was extracted for analysis.

For analysis, 100 µL of sample extraction solution (blank control), standard solution, or sample was added to a microplate, gently mixed, and incubated in a light-avoiding environment at 25 °C for 45 min. Subsequently, the liquid in the wells was discarded, and each well was thoroughly washed 4–5 times with 250 µL of washing solution per well, with a 10-s interval between each wash. After patting dry with absorbent paper, 100 µL of antibody working solution was added to each well, gently mixed, and then incubated at 25 °C in a light-avoiding environment for 30 min. Subsequently, the plate underwent washing again 4–5 times, and 100 µL of enzyme working solution was added to each well, followed by gentle mixing and incubation under the same conditions for 30 min. After repeating the washing step 4–5 times, 100 µL of chromogenic reagent was added to each well, and the plate was incubated at 25 °C in a light-avoiding environment for 15 min. Finally, 100 µL of stop solution was added to each well and gently mixed, and the OD values for each well were measured at 450 nm using a microplate reader.

A Cry1C transgenic protein rapid test kit (Youlong Biotech, Shanghai, China, Cat# AA0541) from Youlong Corporation was used to generate a Cry1C standard curve. The curve was determined as y = 0.2416x + 0.2437, with an R² value of 0.9958, following the instructions of the manufacturer.

### 4.9. Statistical Analysis

Statistical analysis was conducted using SPSS 26.0.0.0, while GraphPad Prism 8.4.3 was used to visualize the data. Significant differences between the means were determined using the least significant difference test (*p* < 0.05) and two-sided Student’s *t*-test (*p* < 0.05).

## Figures and Tables

**Table 1:** Evaluation of SPB resistance of transgenic lines through T1 to T3 generations in field conditions. WT, receptor variety Kennong18; KC1, KC3, and KC8, transgenic lines; T1, T2 and T3, transgenic generation 1, 2, and 3; a, b and c indicate significant differences compared with WT (*p* < 0.05).

Name of Lines	T1	T2	T3
WT	11.65% ± 2.21% c	14.51% ± 1.65% c	13.27% ± 1.92% c
KC1	0.31% ± 0.02% a	1.72% ± 0.45% a	0.54% ± 0.09% a
KC3	3.69% ± 0.71% b	4.52% ± 0.78% b	2.71% ± 0.53% b
KC8	2.94% ± 0.94% b	3.87% ± 0.92% b	4.18% ± 0.64% b

**Table 2:** Agronomic and quality traits of transgenic soybean lines. Different lowercase letters following data in the same column indicate significant differences at the 0.05 level.

Name of Line	Plant Height	Branch Number	Pod Number	Seed Number	100 Seed Weight (g)	Protein Content (%)	Oil Content (%)
WT	70.5 ± 8.8 a	5.7 ± 1.8 a	83 ± 14.2 a	155.4 ± 27.6 a	11.2 ± 1.8 a	41.1 ± 0.32 a	22.1 ± 0.16 a
KC-1	70.3 ± 13.1 a	4.3 ± 1.2 a	66 ± 18.7 a	134.8 ± 29.8 a	11.0 ± 3.4 a	41.8 ± 0.15 a	22.4 ± 0.09 a
KC-3	73.6 ± 6.1 a	4.1 ± 2.2 a	77 ± 10.7 a	126.1 ± 19.5 a	12.4 ± 2.3 a	40.1 ± 0.51 a	22.8 ± 0.12 a
KC-8	74.5 ± 5.4 a	4.6 ± 1.9 a	74 ± 19.8 a	128.2 ± 15.9 a	11.9 ± 2.9 a	41.3 ± 0.31 a	22.5 ± 0.25 a

**Table 3:** The cry1c* protein level in different tissues. Different lowercase letters following data in the same row indicate significant differences at the 0.05 level.

Developmental Stages	μg/g of Fresh Weight					
	Root	Shoot	Leaf	Flower	Pod	Seed
V2	0.26 ± 0.15 a	1.51 ± 0.03 b	4.81 ± 0.43 c	-	-	-
R3	0.26 ± 0.06 a	6.05 ± 2.68 b	3.15 ± 0.14 b	0.26 ± 0.04 a	18.4 ± 0.81 c	-
R6	2.74 ± 0.18 a	8.53 ± 0.67 b	5.22 ± 0.39 b	-	-	14.74 ± 0.67 c
R8	0.67 ± 0.19 a	0.67 ± 0.04 a	12.26 ± 0.8 b	-	-	12.43 ± 0.66 b

## Data Availability

The original contributions presented in the study are included in the article/Supplementary Material, further inquiries can be directed to the corresponding authors.

## Supplementary Materials

The following supporting information can be downloaded at: https://www.mdpi.com/article/10.3390/plants13050630/s1, Figure S1: In-planta feeding bioassays of *S. litura*; Figure S2: In-planta feeding bioassays of *M.separta*; Figure S3: Western blot analyses for the detection of *cry1c** in the tissues of the transgenic line KC1 are shown. The positive control was the Cry1C protein provided by Youlong Biotechnological Co., Ltd. The negative control consisted of KN18 leaves. Arrows indicate the band of the *cry1c** protein; Table S1: The list of primers used in this study.
