# Supplementary material for: Overexpression of cry1c* Enhances Resistance against to Soybean Pod Borer (Leguminivora glycinivorella) in Soybean
Source: Plants (Basel). 2024 Feb 25;13(5):630. doi: 10.3390/plants13050630 (PMC10933787; doi:10.3390/plants13050630)
Supplement: Supplementary file 1 [file plants-13-00630-s001.zip › plants-2757025-supplementary.pdf]

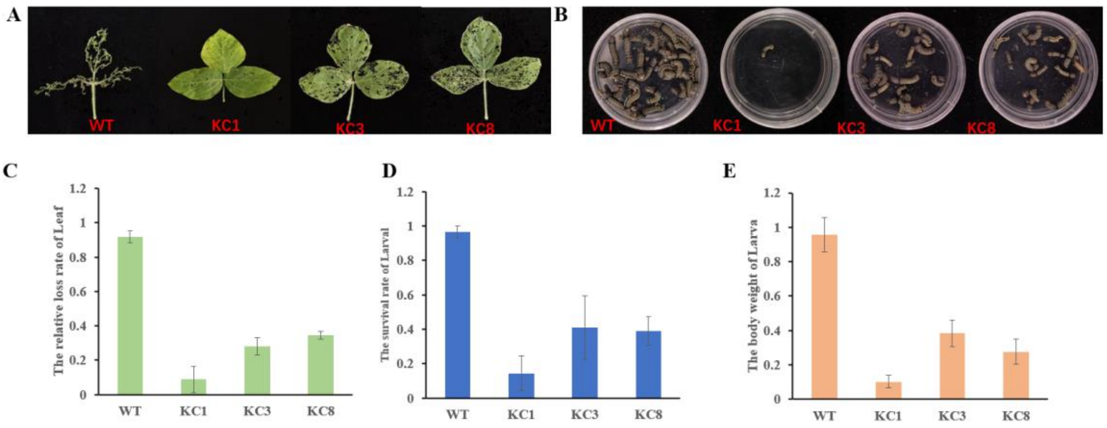

Figure S1. In planta feeding bioassays of *S. litura*. (**A**) The phenotype of feeding situation by *S. litura* larvae; (**B**) Survived larvae of *S. litura* after 15 days feeding; (**C**) The relative loss rate of leaf that fed by *S.* **litura** larvae; (**D**) The survival rate of *S. litura* larvaess; (**E**) The body weight of *S. litura* larvae.

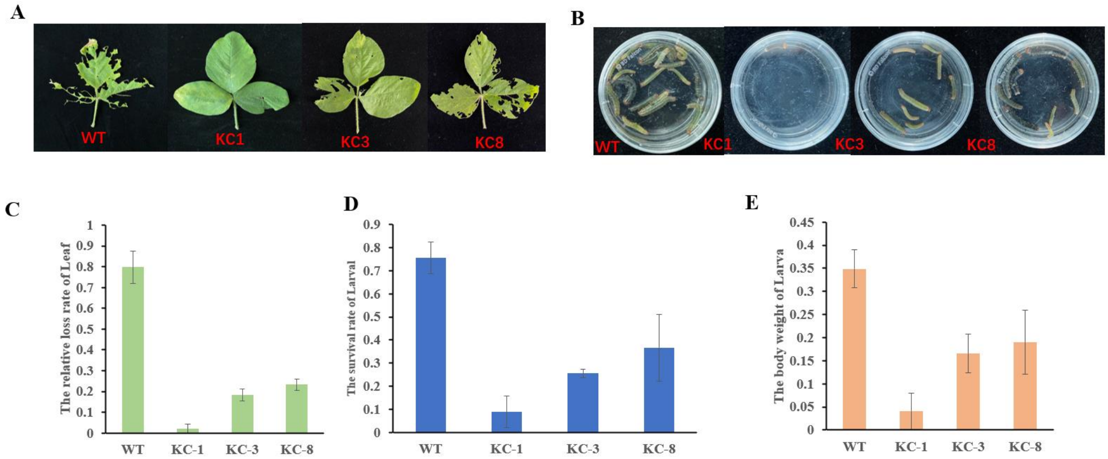

Figure S2. In planta feeding bioassays of *M.separta*. (**A**) The phenotype of feeding situation by *M.separta* larvae; (**B**) Survived larvae of *M.separta* after 15 days feeding; (**C**) The relative loss rate of leaf that fed by *M.separta* larvae; (**D**) The survival rate of *M.separta* larvae; (**E**) The body weight of *M.separta* larvae.

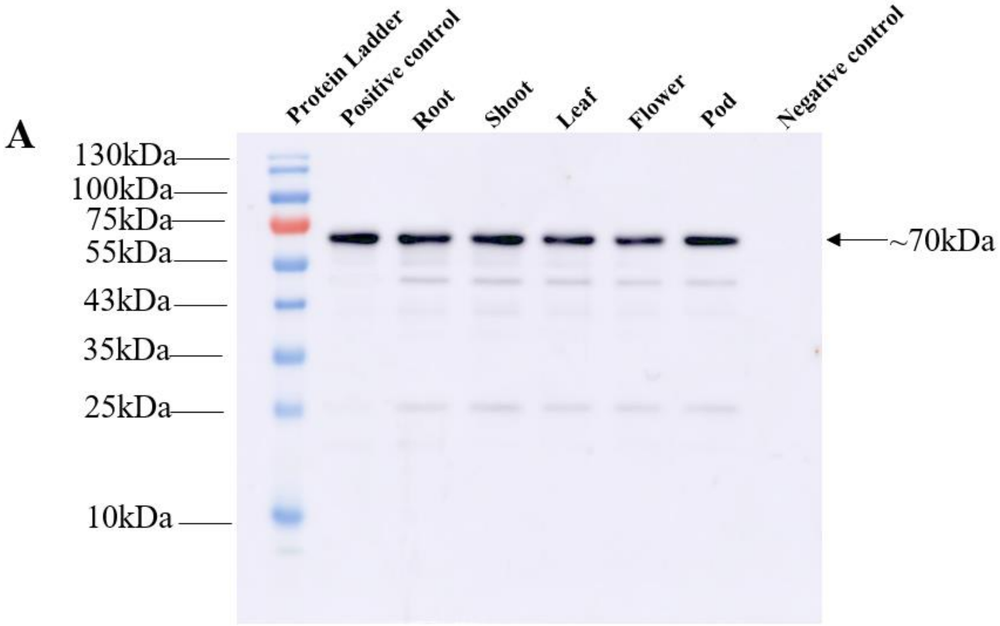

Figure S3. Western blot analyses for detection Cry1C* in the tissues of transgenic line KC1 are shown. Positive control is Cry1C protein provide by Youlong Biotechnological Co., LTD. Negative control is leaf of KN18. Arrows indicate the band of the Cry1C* protein.

Table S1. The list of primers used in this study.

Name of Lines	Sequences
Cry1C -F1	TTCTACTGGGGAGGACATCG
Cry1C -R1	CGGTATCTTTGGGTGATTGG
Bar-F1	GCGGTACCGGCAGGCTGAAG
Bar-R1	CCGCAGGAACCGCAGGAGTG
Act-F	TTGACTGAGCGTGGTTATTCC
Act-R	GATCTTCATGCT GCTGGGTG
Cry1C FA-F1	TCTCTCTCACTTGTTCAGTTCTTGGT-3
Cry1C FA-R1	TGCTGGGTTG TTAGGATCTTCTT-3
KC1GL-F1	TTTTTTTTACCAGAACACCC
BarR1	GGTCAACTTCCGTACCGAGC
